# An intrahepatic Portal Vein Aneurysm Presenting with Esophageal Variceal Bleeding in a Pediatric Patient: A Rare Clinical Entity

**DOI:** 10.4274/balkanmedj.2017.1776

**Published:** 2018-11-15

**Authors:** Şükrü Güngör, Fatma İlknur Varol, Ramazan Kutlu, Sezai Yılmaz, Mukadder Ayşe Selimoğlu

**Affiliations:** 1 Department of Pediatric Gastroenterology, Hepatology, and Nutrition, İnönü University School of Medicine, Malatya, Turkey; 2 Department of Radiology, İnönü University School of Medicine, Malatya, Turkey; 3 Department of General Surgery, İnönü University School of Medicine, Malatya, Turkey

An 11-month-old girl suffering from hematemesis was referred to our hospital. She had a past history of recurrent bleeding that occurred four times, which was accompanied by melena during hospitalization. The patient’s history was unremarkable for trauma, surgical intervention, or liver disease. Physical examination revealed normal findings. There were no ascites, dilated abdominal wall veins, spider nevi, organomegaly, peripheral edema, and finger clubbing that are suggestive of chronic liver disease. Coagulation tests and platelet levels were normal. Upper gastrointestinal endoscopic examination revealed grade 3 esophageal and fundal varices (Figure 1a, 1b). Color Doppler ultrasound revealed a monophasic turbulent venous flow pattern within the intrahepatic portal vein aneurysm. Dynamic computed tomography scans showed fusiform aneurysmal enlargement in the proximal segment of the intrahepatic right portal vein (Figure 2a, 2b). The patient did not respond to medical treatment (propranolol and somatostatin). Endoscopic treatment of fundal varices could not be attempted due to the young age of the patient. Surgical intervention was not considered as the aneurysm was located proximal to the intrahepatic branches of the main portal vein; therefore, the patient underwent the Sugiura operation. Endoscopic examination performed 3 months after the operation showed significant regression of esophageal varices (Figure 3a, 3b). Control dynamic computed tomography scans performed after 6 months revealed not only the persistence of aneurysmal dilation but also thrombosis within the aneurysm and the portal vein (Figure 4a, 4b). Informed consent was obtained from the parents for the publication of the patient’s images.

Although venous aneurysms primarily occur in lower extremity veins, they can rarely be observed in the portal system (1). In a retrospective study, portal vein aneurysm was detected in 0.43% of 4.181 randomly selected individuals, 72% of whom were asymptomatic (2). The same study also reported that the youngest age at diagnosis was 24 years (mean age 53 years), and there was no predilection for sex (2). The present case had no chance of surgery (liver resection) as the aneurysm was situated in the right intrahepatic portal vein in close proximity to the intrahepatic portal vein bifurcation. Percutaneous embolization was not considered as a treatment modality due to the concern of iatrogenic portal vein thrombosis and liver dysfunction as the aneurysm was also proximal to the right portal vein. The Sugiura operation had to be performed because of recurrent bleeding. Gastrointestinal endoscopic examination performed 3 months after surgery showed regression of the esophageal and fundal varices (Figure 3a, 3b). Unfortunately, portal vein thrombosis occurred in the follow-up probably due to venous stasis (Figure 4a, 4b) (3). To our knowledge, there are no studies using large case series and a consensus on the use of anticoagulants in patients with portal vein thrombosis. Anticoagulant therapy was not administered before or after surgery; as a self-critic, anticoagulants might have possibly prevented thrombosis after surgery in our patient. Currently, the patient receives 1 mg/kg/day of propranolol to prevent the recurrence of esophageal varices. Ultrasound and endoscopy are being repeated once every 6 months. In conclusion, intrahepatic portal vein aneurysms may present with massive upper gastrointestinal bleeding even in infancy without any previous symptoms, suggesting liver dysfunction. Intrahepatic portal vein aneurysms, though rare, should not be overlooked in the differential diagnosis of pediatric portal hypertension.

## Figures and Tables

**Figure 1:**
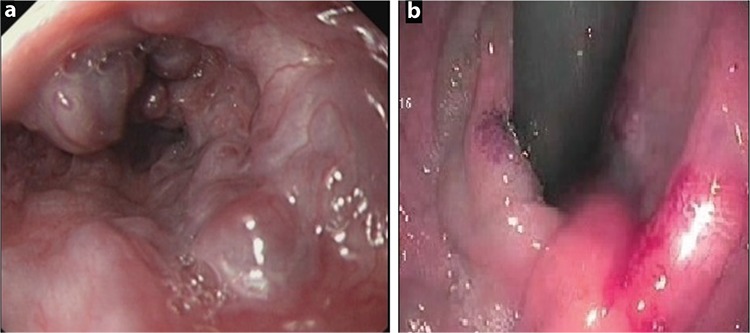
a, b. Endoscopic view of esophageal and fundal varices before operation.

**Figure 2:**
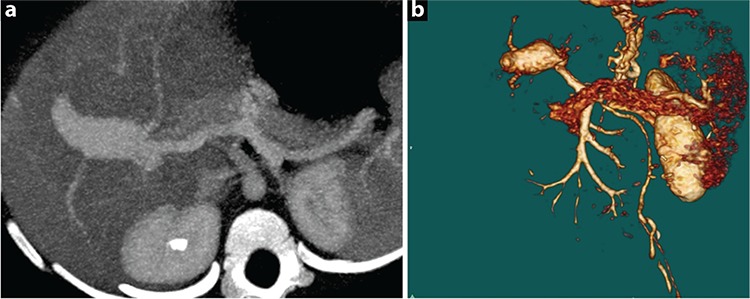
a, b. Preoperative maximal inspiratory pressure (a) and 3D reconstructed dynamic abdominal computed tomography images showing fusiform aneurysmal dilation in the proximal end of the intrahepatic portal vein and prominent esophageal varices (b).

**Figure 3:**
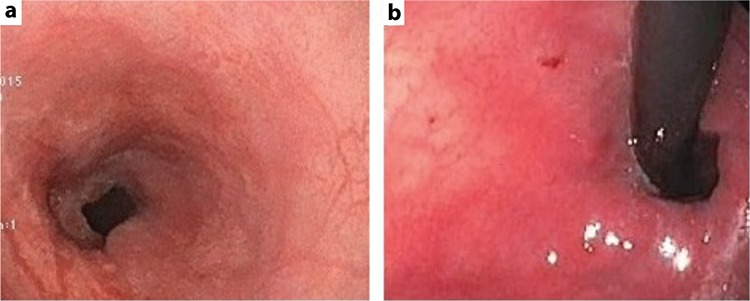
a, b. Endoscopic view of esophagus and fundus after operation.

**Figure 4:**
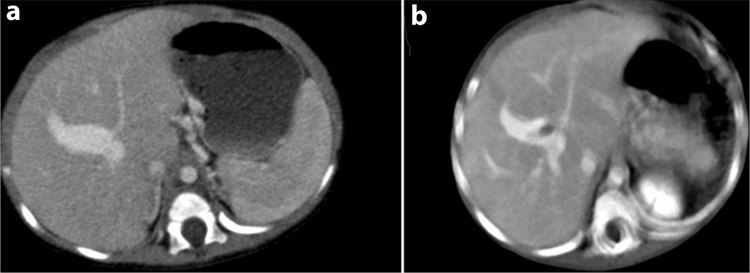
a, b. Preoperative axial computed tomography showing fusiform portal venous aneurysmal dilatation (a), corresponding postoperative axial computed tomography image (b) demonstrates thrombus inside this dilated portal vein.
